# Stretching DNA origami: effect of nicks and Holliday junctions on the axial stiffness

**DOI:** 10.1093/nar/gkaa985

**Published:** 2020-11-05

**Authors:** Wei-Hung Jung, Enze Chen, Remi Veneziano, Stavros Gaitanaros, Yun Chen

**Affiliations:** Department of Mechanical Engineering, Johns Hopkins University, USA; Institute for NanoBioTechnology, Johns Hopkins University, USA; Center for Cell Dynamics, Johns Hopkins University, USA; Department of Civil and Systems Engineering, Johns Hopkins University, USA; Department of Bioengineering, George Mason University, USA; Institute for Advanced Biomedical Research, George Mason University, USA; Department of Civil and Systems Engineering, Johns Hopkins University, USA; Department of Mechanical Engineering, Johns Hopkins University, USA; Institute for NanoBioTechnology, Johns Hopkins University, USA; Center for Cell Dynamics, Johns Hopkins University, USA

## Abstract

The axial stiffness of DNA origami is determined as a function of key nanostructural characteristics. Different constructs of two-helix nanobeams with specified densities of nicks and Holliday junctions are synthesized and stretched by fluid flow. Implementing single particle tracking to extract force–displacement curves enables the measurement of DNA origami stiffness values at the enthalpic elasticity regime, i.e. for forces larger than 15 pN. Comparisons between ligated and nicked helices show that the latter exhibit nearly a two-fold decrease in axial stiffness. Numerical models that treat the DNA helices as elastic rods are used to evaluate the local loss of stiffness at the locations of nicks and Holliday junctions. It is shown that the models reproduce the experimental data accurately, indicating that both of these design characteristics yield a local stiffness two orders of magnitude smaller than the corresponding value of the intact double-helix. This local degradation in turn leads to a macroscopic loss of stiffness that is evaluated numerically for multi-helix DNA bundles.

## INTRODUCTION

**Figure 1.**
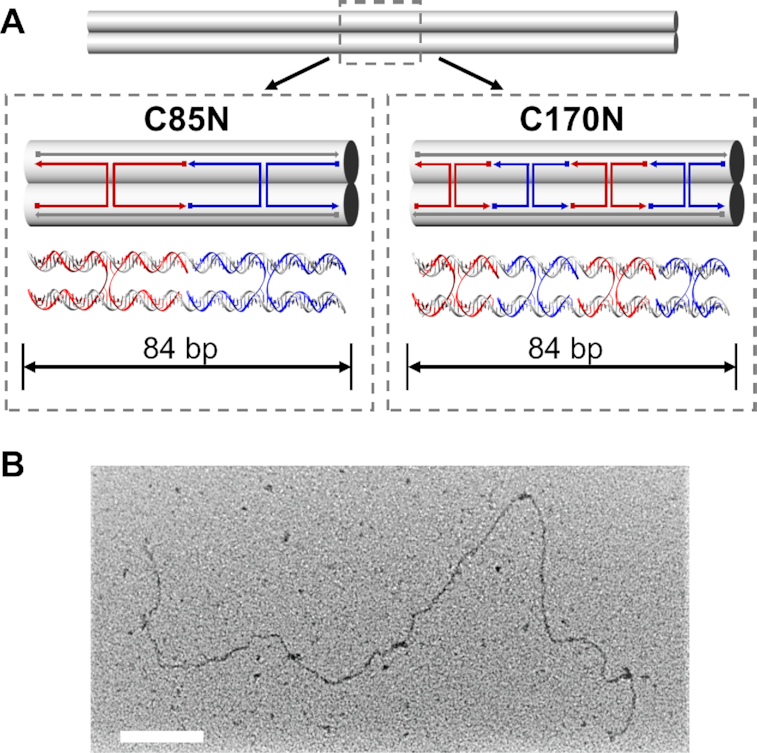
DNA nanobeams constructs with prescribed nick and HJs densities to be subjected to stretching using hydrodynamic forces. (**A**) DX-based designs of two-helix beams. In construct C85N a double crossover was placed every 42 nucleotides (85 HJs total) while in C170N the crossover occurred every 21 nucleotides (170 HJs total). ▪ indicates the 5′ end and ▸ indicates the 3′ end of the DNA strand. (**B**) TEM images showed that the length of the nanobeams was approximately 1.2 μm, as expected from the design. Scale bar: 100 nm.

The controlled self-assembly of synthetic DNA strands offers the unique ability to build complex 2D and 3D architectures with nanoscale precision (1–4). These DNA nanostructures can serve as scaffolds for positioning both organic and inorganic molecules, thus having tremendous potential in many areas including drug delivery, super-resolution imaging and nano-manufacturing (5–11). The two main approaches for the synthesis of DNA nanostructures are connecting small unit blocks (‘tiles’) and the DNA ‘origami’ method. The DNA origami method involves folding one long single strand of DNA (ssDNA), the ‘scaffold’ strand, into different desired shapes by locally hybridizing short oligonucleotides known as ‘staple’ strands (12). Typically, the scaffold strand is derived from the M13mp18 bacteriophage and has an approximate length of 7k nucleotides while optimal synthesis and robustness dictates the length of the staples to be between 15 and 60 nucleotides (13). In between consecutive staple strands’ 5′ and 3′-ends there are small segments of single-stranded DNA (ssDNA) that correspond to discontinuities of the backbone, known as nicks (see gaps between the red and blue strands in Figure 1A). The absence of a phosphodiester bond between these adjacent nucleotides to covalently connect the two staples can affect the mechanical properties of the helix (14).

A large number of nanostructures with a plethora of different shapes and sizes, ranging from 10 nm to several microns, have been created using the DNA origami approach (15–17). Immobile Holliday junctions (HJs) are crossovers that connect neighboring DNA helices are the main motif of all DNA nanostructures (2). One can combine two HJs to create a double crossover motif (DX-tile) (18), which significantly increases the rigidity of DNA nanostructures and has thus been the key building block in many self-assembled scaffolds, both origami- and tile-based ones (19–22). The distance between consecutive HJs in the DX-tile is typically chosen to be 2*n*Z nucleotides, where *n* is the period of B-form DNA (10.5 nucleotides) and *Z* is an integer. This choice leads to minimal distortions that can arise from the development of internal bending and torsional moments.

Although there have been numerous works that focus on the self-assembly conditions, aiming at an increased yield and stability of the synthesized nanostructures, the mechanical properties of the resulting assemblies have been relatively unexplored, with the exception of their bending resistance and the corresponding persistence length (23,24). The mechanical behavior of DNA origami is derived by a complex interplay among several factors, such as the mechanics of single- and double-stranded DNA (dsDNA), the number and location of HJs, the local properties of nicked helices, as well as the effect of external environmental conditions (25–27). Desired nanostructures with complex geometric shapes and/or dynamical behavior can be achieved by tuning these parameters (15,28–32). In select studies, the mechanics of DNA nanostructures under external forces/torques were examined to estimate their stiffness (33–35) and improve the robustness of the designed assembly (36–38). A limitation in most of the results reported to date, however, is that they typically concern specific DNA assemblies, making it difficult to connect their properties to the underlying nanostructural characteristics. Design efforts thus resort to over-engineering, i.e. increasing the number of helices in each origami member in order to achieve sufficient mechanical performance (36–38). These ad-hoc methodologies, however, not only result in unnecessarily increased complexity, but often to counter-intuitive mechanical behavior (38).

Here, we systematically design DX tile-based nanobeams with controlled characteristics, and subsequently synthesize and test them to extract their mechanical properties under tensile forces. By varying key design parameters, such as the density of HJs and nicks, we are able to measure experimentally their uncoupled effect on the resulting axial stiffness of DNA nanobeams. Coarse-grained continuum modeling and the finite element method is used to reproduce the experimental data and further examine the stiffness of DNA origami beams. Constructs with a varying number of helices (2–10) stacked in different packings, i.e. both honeycomb and square, are tested *in silico* and their relative force–displacement curves are calculated. The apparent stiffness of each beam is reported and compared to theoretical estimates that assume that helices are rigidly connected and ignore the effect of nicks.

## MATERIALS AND METHODS

### DNA origami assembly

DNA staple strands were purchased from IDT and used directly for folding. The M13 Bacteriophage single-stranded DNA was used as scaffold for origami construction. In a typical sample preparation, staple strands were mixed with the ssDNA scaffold (M13mp18 7249 from Guild Biosciences) at 10 nM final concentration in 10-fold molar excess (100 nM) in folding buffer (5 mM Tris base, 1 mM EDTA, supplement with 14 mM MgCl_2_) in a total volume of 50 μl. The solution was slowly annealed from 95°C down to room temperature (24°C) in a PCR thermal cycler overnight using the following program: 95°C for 5 min, 80° to 70°C at 1°C per 5 min, 70° to 30°C at 1°C per 15 min and 30° to 25°C at 1°C per 10 min. Note that for the purpose to conjugate the DNA nanobeams to the surface of the glass chamber via anti-digoxigenin antibody and to the particle coated with streptavidin, the staple strands positioned at both ends of the DNA nanobeams were conjugated with either biotin or digoxigenin.

### Ligation of consecutive DNA staple strands

The protocol used for ligation of the DNA origami constructs was adapted from Wang *et al.* (39). Briefly, after annealing and purification of the nanostructures with the Biorad PCR Kleen purification system to remove the excess of staple strands, the nanostructures were incubated with the T4 Polynucleotide Kinase to ensure phosphorylation of all 5′-ends. The incubation was performed at 37°C for 4 h in the provided T4 ligase buffer. After the 4 h of incubation, T4 DNA ligase was added to the mix to ligate all nicks of the structures and the sample were incubated at 37°C for an extra hour.

### Gel electrophoresis

Folded DNA origami constructs were subjected to 1.5% native agarose gel electrophoresis and run it using 70V for 2 h (gel prepared in 0.5× TBE buffer supplemented with 11 mM MgCl_2_ and 0.005% (v/v) EtBr) in an ice water bath. The image was acquired with an Azure C150 gel documentation system (Azure Biosystems). Following the gel electrophoresis, the target gel bands were excised and placed into a Freeze ’N Squeeze column (Bio-Rad Laboratories, Inc.). The excised gel was crushed into fine pieces by a microtube pestle in the column, and the column was then centrifuged at 7000 g for 5 min.

### TEM preparation

The DNA origami construct (C170L) with concentration of 10 ng/μl was used for TEM imaging. Two microliters of the sample were adsorbed for 2 min onto plasma treated carbon-coated TEM grids. The grids were then stained for 10 s using a 1% aqueous uranyl acetate solution and followed by three times 10 s washing using deionized water and letting it to air-dry. Imaging was performed using a FEI F200C Talos 200 keV FEG transmission electron microscope.

### Flow chamber preparation

Flow chambers were made by bonding air-plasma treated polydimethylsiloxane (PDMS) (Dow Corning, Sylgard 184) slabs to 22 × 50 mm cover glass (Corning, 2975–225); the PDMS slabs were cast over scotch tape strips (3M, Scotch^®^ Magic™ tape 810) to form shallow channels with dimension of 15 mm × 1 mm × 0.06 mm (length × width × height, respectively). Inflow and outflow ports were formed by puncturing the PDMS with harris punch (Harris Uni-Core I.D. 4 mm). To immobilize the DNA origami, flow chambers were manually filled with 0.1 μM polyclonal sheep anti-digoxigenin antibody (Sigma-Aldrich, 11333089001) in phosphate buffered saline (PBS) (Thermo Fisher Scientific, 10010049) and incubated for 2 h to non-specifically adsorb anti-digoxigenin antibody on the glass surfaces. Flow of PBS, with a rate of 50 μl/min for 30 min, was then used to wash out the antibody. After manually removing the PBS in the chamber, the blocking solution with 3 mg/ml bovine serum albumin (Sigma-Aldrich, A9647) and 0.1% Tween 20 (Promega, H5152) in PBS was then manually added into the flow chambers and incubated for additional 2 h. The blocking solution was then manually removed and the DNA beam solution with concentration of 100 ng/μl was added in the flow chambers and remained overnight at 4°C. Subsequently, the DNA solution in the flow chamber was removed, and PBS was manually added to the chamber and incubated for 2 h. Lastly, 20 μl of the 1 μm streptavidin coated fluorescent particles (Bangs Laboratories, Dragon Green, CFDG004) at a density of 4.7 × 10^9^ particles/ml were added to each channel and incubated for 2 h. According to the vendor, 97.6% of the fluorescent particles have a diameter ranging from 0.95 μm to 1.05 μm.

### Imaging acquisition

The displacement of the DNA nanobeam-tethered particle under flow was recorded in timelapse images using a fluorescence microscope (Leica TCS SP8) with a 63X objective with an oil-immersion lens, and with a CCD camera (Leica DFC365 FX) , at the sampling rate of 5 frames/s. The image size of each frame was 1392 pixels ×  1040 pixels. A Xenon arc lamp (Leica EL6000) with a FITC filter set (480/40 nm band-pass excitation filter, 527/30 nm band-pass emission filter, and 505 nm dichroic filter) were used to visualize the fluorescence of the tethered particles.

### Axial stiffness measurement

A constant flow rate of 100–1300 μl/min, generated by a syringe pump (NE-1002X, Pump System Inc.) and resulting in approximately 5–65 pN particle drag force, was utilized to create hydrodynamic forces that stretch the DNA nanobeams. The elbow Luer connector male (Ibidi, 10802) and tube adapter set (Ibidi, 10831) were then used to connect flow chambers to syringe pump. The particles attached to the DNA nanobeams were imaged at 0.2 s interval for 3.5 min. The particle coordinates were tracked in order to measure their displacement using TrackMate (FIJI-ImageJ, NIH) (40), and subsequently used to evaluate drag forces at the interface experienced by the particles, as described in (41). Briefly, the force applied on the particle was estimated by Stokes’ law as $F\ = \ 6\pi {\rm{\eta rv}}$, where η is the dynamic viscosity, *r* is the radius of the particle and *v* is the flow velocity. The flow velocity profile is given by ${v_x}\ ( z ) = \ 4{v_{max}}\ \frac{z}{h}( {1 - \ \frac{z}{h}} )$, where *h* is the height of the channel, *z* is the distance between the centroid of particle from the glass surface, and *v*_max_ is the maximum velocity of the flow profile (41). *v*_max_ can be obtained from the formula ${v_{max}} = \frac{{3Q}}{{2wh}}$, where *Q* is the volume flow rate and *w* is the width of the channel.

To ensure that only particles optimally tethered to the DNA were analyzed, we applied flow in both directions to examine the corresponding displacement of the particles. The time-displacement curve for each particle was plotted for inspection, where the displacement was defined as the absolute value of the difference between the initial position and the current position. The particles which did not exhibit symmetric displacement after the flow direction was reversed, mirroring the displacement under forward flow, were then excluded for further analysis. For the remaining particles, we proceeded to estimate the force response, by first obtaining the unstretched length of the DNA origami. This value is calculated as the position difference of the particle before the flow was applied and when 10-pN hydrodynamic force was applied, at which point the DNA origami was straightened but not stretched. As the applied hydrodynamic force exceeded 10 pN, the extension of the DNA origami was recorded as the particle was further displaced by the flow, stretching the DNA origami. The slope of the applied force over the displacement was then determined by linear fitting to obtain the value of the axial stretching stiffness.

## RESULTS AND DISCUSSION

### Design of DNA nanobeams with tailored densities of Nicks-HJs

To evaluate the effect of nicks and HJs on the axial stiffness of DNA nanostructures, we used two designs of DNA origami nanobeams that were synthesized and then modified to produce four different constructs. All nanobeams consisted of two DNA duplexes connected through HJs, creating a series of periodic DX-tiles. The first design, C170N, had a double-crossover every 21 nucleotides (170 HJs total), while in the second design, C85N, the distance between successive HJs was set to be 42 nucleotides (85 HJs total) (Supplementary Figure S1). The crossover spacing is chosen to ensure that the beams have negligible pre-strains caused by under- or over-twisting. Note that both these HJs densities create a domain gap between neighboring helices, mainly caused by electrostatic interactions, but this effect is eliminated under large stretching forces. In both designs, shown in Figure 1A, the two helices were nicked in each center between consecutive crossovers, resulting in their total number of nicks being 340 and 170 respectively. Using T4 DNA ligase in both constructs leads to two more nanobeams, namely constructs C170L and C85L, which had all nicks ligated and the same crossover densities as the C170N and C85N correspondingly. This allowed us to evaluate the uncoupled effects of HJs and nicks by measuring the stretching stiffness of each DNA nanobeam. To examine the ligation efficiency, we have performed qPCR to compare the melting temperature of C85N and C85L nanobeams. Nicks between the staple DNA strands are the likely locations where dehybridization, also known as melting, is initiated. More nicks result in lower melting temperature of the DNA nanobeams. A significant shift in the melting temperature from 76°C (C85N) to 80.5°C (C85L) was observed (Supplementary Figure S2). The higher temperature required to dehybridize the double helix DNA in C85L indicates the ligase successfully formed the phosphodiester bond between adjacent staple DNA strands. Moreover, we also observed a broadening of the peak for the C85L structure (Supplementary Figure S2), corresponding to the higher thermal stability when nicks are ligated as previously reported (42). All constructs were synthesized using a one-pot annealing process overnight (12,13). The simplicity of the designs guarantees a synthesis process with very high yield, which for single-layer DNA origami is typically close to 100% (12,13). The successful assembly was confirmed by gel-electrophoresis (Supplementary Figure S3) while transmission electron microscopy (TEM) imaging (Figure 1B) verified that the length of the synthesized nanobeams was approximately 1.2 μm, corresponding to half the length of the single-stranded M13mp18 scaffold. Given the reported persistence length for dsDNA is in the range of 40–60 nm (43,44), DX-tiles show a two-fold increase of that (18), and for four helix-bundles the persistence length is in the range of 600–800 nm (33), the total length (1.2 μm) of our DNA origami exceeds the value of its persistence length, thus making it flexible at its unstretched state. Such flexibility can be readily observed in the TEM image (Supplementary Figure S4).

### Stretching experiments and axial stiffness measurements

**Figure 2.**
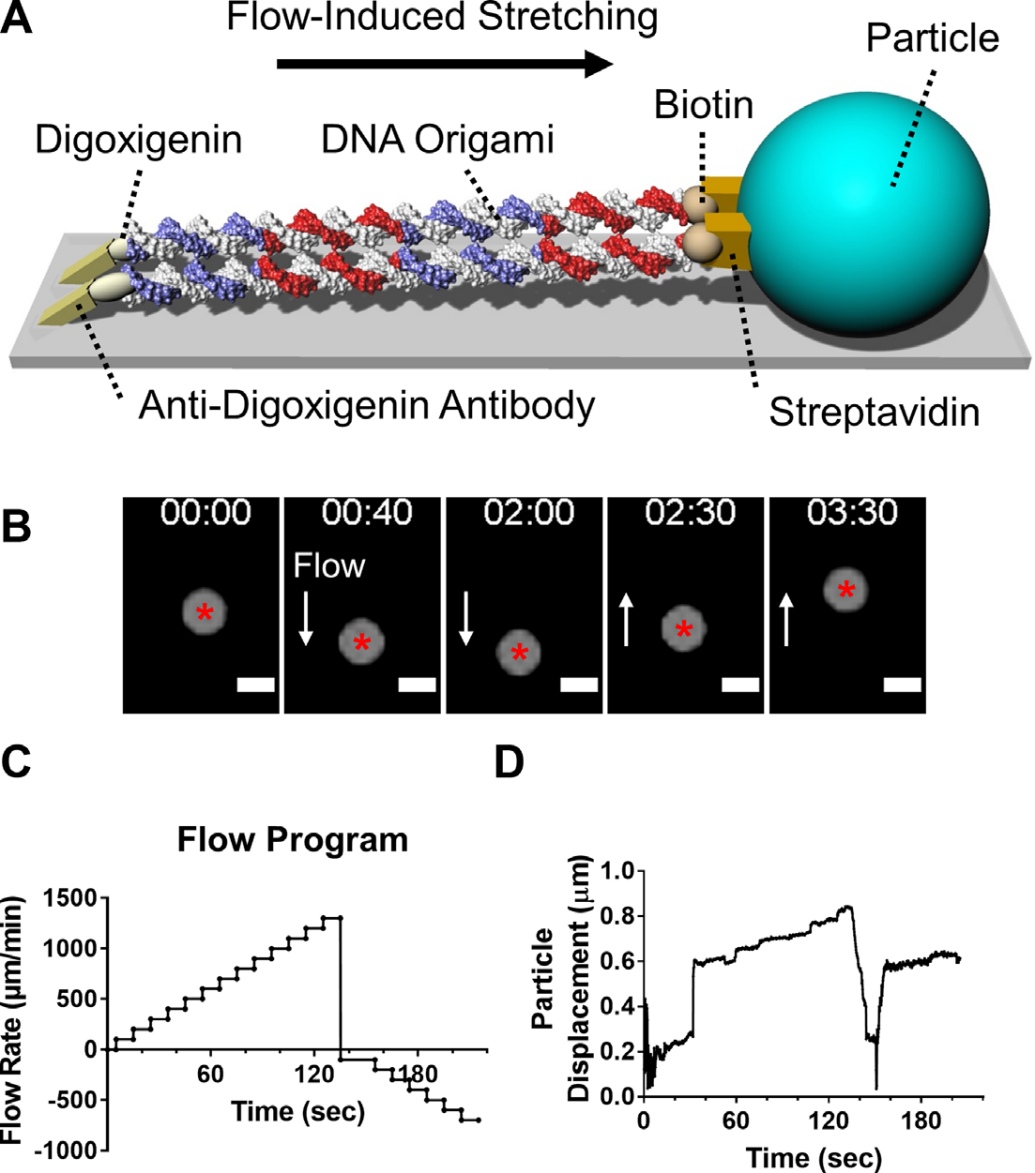
Flow-induced stretching of DNA origami and particle tracing set-up. (**A**) The experimental scheme for the axial stretching of the DNA nanobeams using microfluidics is shown. One end of the nanobeam was conjugated with digoxigenin and then immobilized on an anti-digoxigenin antibody-grafted glass surface. The free end of the DNA nanobeam was conjugated with biotin and then bound to a streptavidin-coated, micron-sized particle. The stretching force was applied hydrodynamically through the particle by flowing the buffer through the microfluidic device at specified rates. The length of the DNA origami is approximately 1.2 μm, and the diameter of the particle is 1 μm. (**B**) Representative timelapse images show the displacement of the micron-sized particle bound to the DNA nanobeam by the flow. The XY coordinate of the centroid (red asterisk) was recorded as the DNA nanobeam stretched along the direction of the flow (arrow) at various rates, imposing different magnitudes of stretching forces. Timestamp format is mm:ss. Scale bar: 1 μm. (**C**) Flow rates ranging from 100 to 1300 μl/min, generated by a syringe pump exert approximately 5–65 pN as the particle drag force, were used to stretch the DNA nanobeams from 0 sec to 135 sec; and flow rates ranging from 100 to 700 μl/min were applied from 135 sec to 215 sec to stretch the DNA nanobeams in the reversed direction. (**D**) A representative particle displacement is plotted against time for the C170L nanobeam-bound particle. The displacement is defined as the difference between the initial position and the current position.

The axial stiffness measurement of the DNA nanobeams was performed using a microfluidic platform (45), which is an established method to probe the mechanical properties of DNA molecules (39,45,46) as an alternative to optical tweezers. Prior to the measurement, the DNA nanobeams were first immobilized on an anti-digoxigenin antibody-grafted glass surface by one of its extremities (two unhybridized ‘linker’ sequences, see Supplementary Figure S1) conjugated with digoxigenin molecules. The stretching force was applied hydrodynamically through a micron-sized particle bound to the free end of the DNA nanobeam (Figure 2A and B), while the buffer is flown through the microfluidic device. The binding between the particle and the DNA nanobeam was facilitated by the streptavidin-biotin interaction. By adjusting the flow rate (Figure 2C), the stretching force magnitude exerted on the DNA origami can be modulated ranging from 1 pico Newton (pN) to 50 pN (39,45,47). The stretching force was applied step-wise, with 5 pN as the starting magnitude and a 5 pN unit increment every 10 s, until reaching a magnitude of 65 pN. The displacement of the micron-sized particle was tracked (Figure 2D) and the elongation of the DNA origami as a function of the particle displacement was recorded (Movie 1). The force-displacement curves *F–δ* for each DNA origami construct was plotted, the slope was extracted and the axial stiffness *k* was calculated (slope of the curve multiplied by the origami length). Since this study focused only in the apparent axial stiffness of DNA origami past the entropic elasticity regime, we measured the slopes for forces larger than 15 pN. At these force levels, the DNA nanobeams are fully straightened and stretched beyond their original length. To avoid the possible impact of ethidium bromide (EtBr) staining on the mechanical properties of DNA nanobeams, we used trace amount of EtBr (< 0.03 μg/ml) to stain the DNA before gel extraction. After gel extraction and subsequent washing, we could not observe EtBr fluorescence during the stretching experiment (Figure 2B). Comparable amount of EtBr was also used in Smith *et al.* (48) for visualization of dsDNA with no effect on the elasticity of the helix. Though Dikic and Seidel (49) showed that the mechanical properties of DNA can be altered when exposed to approximately 400 μg/ml or higher concentration of EtBr, this concentration is at least four orders of magnitude higher than the one retained in the DNA origami synthesized here.

**Figure 3.**
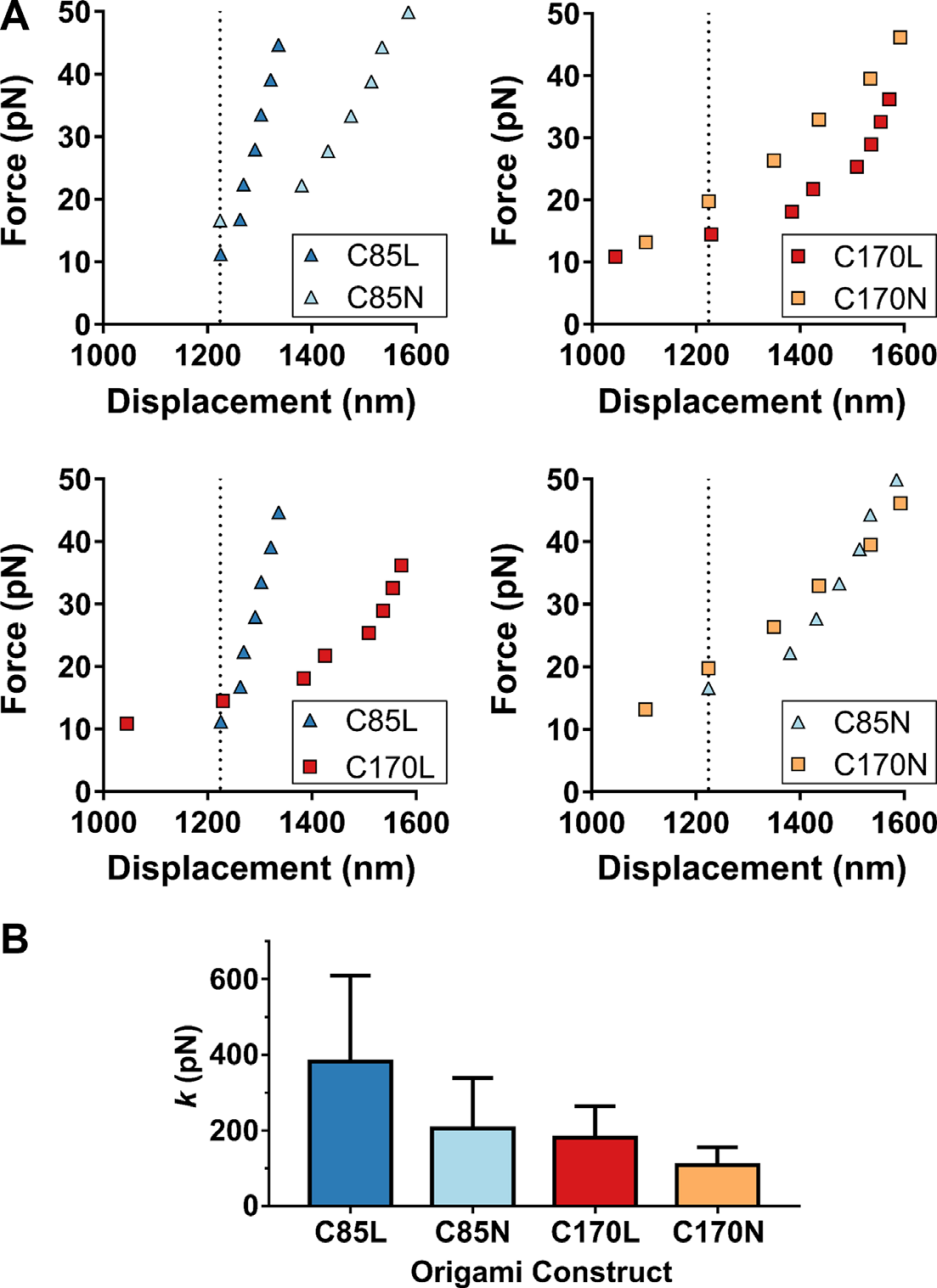
The presence of nicks and HJs reduced the axial stiffness of DNA nanobeams. (**A**) Representative force-displacement responses of constructs C85L, C85N, C170L and C170N are compared. Four combinations of nanobeam pairs are plotted to illustrate how nicks and HJs reduce the apparent stiffness. (**B**) Axial stiffness (*k*) values of C85L, C85N, C170L and C170N are compared. Construct C85L, the ligated nanobeam having the least HJs was the stiffest among all four constructs. C85L: *N* = 5, C85N: *N* = 5, C170L: *N* = 7, C170N: *N* = 4. The error bar shown in the graph represents standard deviation.

Typical responses from four measurements with values very close to the collective mean of each DNA construct are shown in Figure 3A and every experimental replicate in Supplementary Figure S5. The two plots on the top show the difference between the responses of ligated and nicked assemblies with the same number of HJs respectively (85 and 170). The two plots at the bottom showcase the effect of the HJs density on the response of origami with (left plot) and without (right plot) the presence of nicks. The collective results showed that the stiffest response corresponded to the ligated nanobeam with the smaller number of HJs (C85L) while the more compliant beam was the nicked origami with the highest density of HJs (C170N). The mean values and standard deviations of the axial stiffness measured from all the responses of the C85L, C85N, C170L and C170N DNA origami beams are listed in Table 1 (see Figure 3B). We observed an 80% increase of axial stiffness for the ligated nanobeams compared to the corresponding values of their counterparts with the nicked helices. This comparison illustrates that the increased number of nicks significantly reduces the stiffness of the origami. Furthermore, we also observed that reducing the number of HJs by 50% resulted in a near twofold increase of the origami stiffness. This counter-intuitive effect indicates that even though by adding HJs in DNA nanostructures one expects a significant increase in their bending rigidity, it also comes with a substantial decrease of their stretching stiffness. This loss of stiffness was examined further using continuum modeling and finite element simulations corresponding to the same designs tested experimentally.

**Table 1.** The measurement and model prediction values of axial stiffness for DNA nanobeams with different HJs and nicks

Construct	Length (nm)	# HJs (# / 21bp)	# Nicks (# / 21bp)	Mean stiffness ± SD (pN)	Model result (pN)
C85L	1224	85 (0.5)	0 (0)	382 ± 227	328
C85N	1224	85 (0.5)	170 (0.5)	206 ± 131	205
C170L	1224	170 (1)	0 (0)	181 ± 82	181
C170N	1224	170 (1)	340 (1)	108 ± 47	108
6HB (Pfitzner *et al.*)	428	336 (2)	168 (0.5)	337	239
10HB (Pfitzner *et al.*)	257	360 (2)	180 (0.5)	341	409

### Modeling and virtual stretching of DNA origami

To further examine and elucidate our experimental measurements we used computational models that treat each helix as a continuum elastic rod with the effective geometric and material properties of B-form DNA. The sequence-based representations were parsed into finite element models using a CanDo source code that translates each base-pair into a two-node beam element with elastic properties (stretching, bending and torsional stiffness) corresponding to B-form DNA (13,50,51). All crossovers are assumed to rigidly constrain the neighboring helices. The beam elements corresponding to the locations of the HJs and the nicks are assigned axial stiffness values equal to α*k* and β*k* respectively, where *k* is the stretching stiffness of the (intact) double helix and α, β are constants to be determined. In essence, the two constants represent an effective local degradation of the axial stiffness at the locations of crossovers and nicks. We used the finite element software ABAQUS (Simulia) to apply external axial forces on each origami structure and numerically generate their force-extension curves. The nanostructural designs chosen here, i.e. two-helix beams with crossovers every 21/42 nucleotides, lead to a minimal effect from bending and twisting of the DNA helices during stretching, which in other DNA assemblies can be significant.

**Figure 4.**
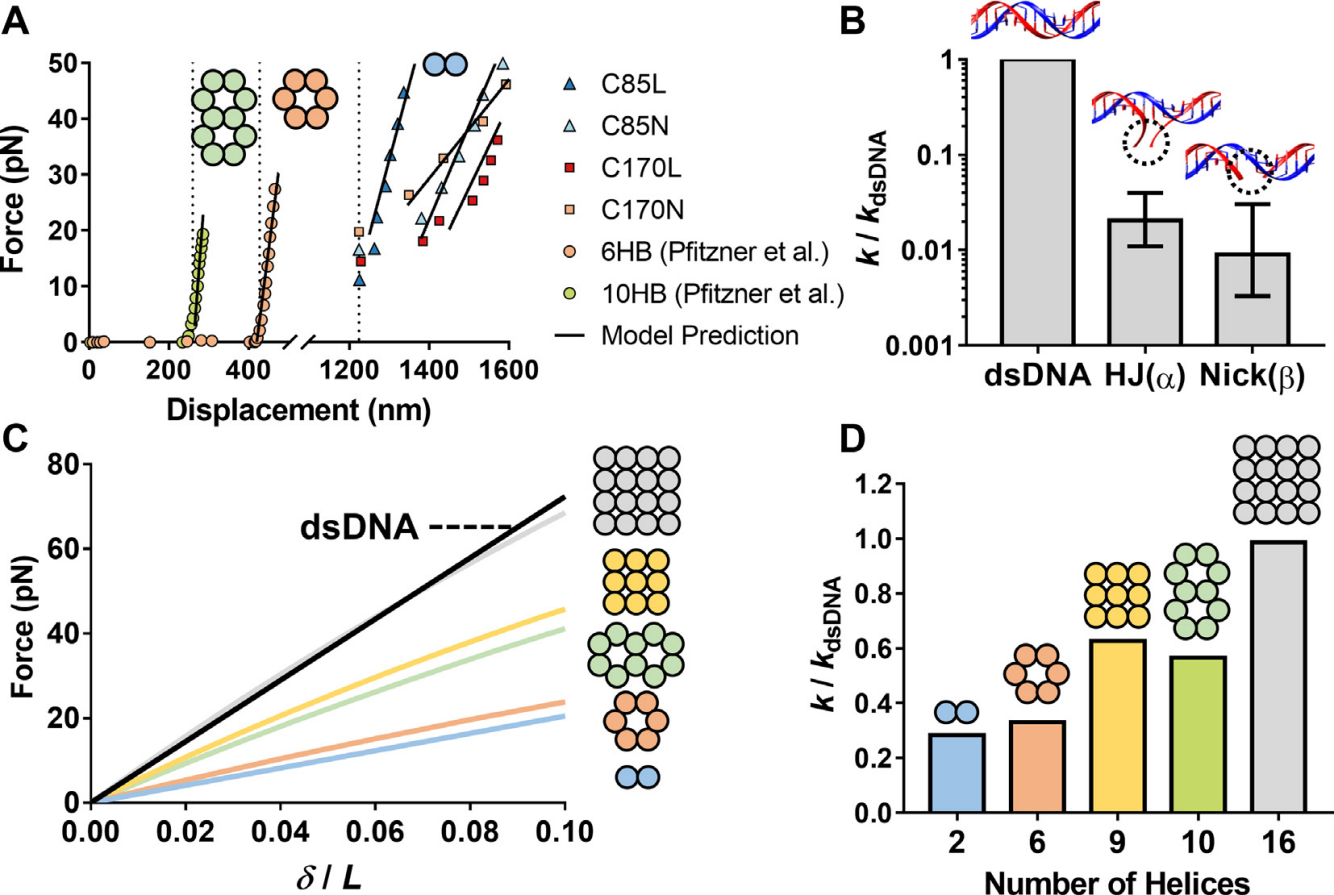
Continuum modeling predicted force-displacement responses agreeing with experimental measurements and showed how the stiffness of multi-helix bundles compares to the one of the double helix. (**A**) Model predictions plotted against the measurements corresponding to constructs C85L, C85N and the 6-helix and 10-helix nanobeams reported in Pfitzner *et al.* (38) showed excellent agreement. (**B**) Local stiffness values at the locations of HJs and nicks are compared to the intact double helix. Optimized fitting showed a corresponding local stiffness for HJs and nicks being 0.0205- and 0.0090-fold respectively of the value of intact B-form DNA. The error bars shown in the graph indicate the confidence intervals. (**C**) The force-displacement curves from tensile simulations on origami beams with cross-sections consisting of two, six, nine, ten, and sixteen helices, representing by blue, orange, yellow, green, and gray lines, respectively. (**D**) The stiffness values for the five origami beams simulated in (C), normalized to the stiffness of B-form DNA, and shown as a function of the number of helices in each packing.

The magnitude of the local stiffness parameter α was first determined by calibrating the numerical response using the average value of the measurements from construct C170L, which does not have any nicks. The same process was then followed using the measurements of construct C170N in order to estimate the local nick stiffness β, keeping the value of the HJ stiffness α constant. Subsequently, using the calibrated parameters, we simulate the force-extension curves for the remaining two nanobeams (C85L and C85N). The results of the numerical simulations, shown in Figure 4A and also reported in Table 1, agree extremely well with the corresponding experimental data. To further examine the validity of our numerical framework and its applicability to different origami designs we also simulated the stretching of a 6- and a 10-helix DNA origami, with approximate lengths of 428 and 257 nm respectively, for which experimental results under tension were reported by Pfitzner *et al.* (38). The predicted response from our simulation, also shown in Figure 4A and reported in Table 1, agreed with the measurements made by Pfitzner *et al.* as well, in spite of the different experimental apparatus used in their work.

The optimal-fit process described above resulted in α = 0.0205 which produced a numerically calculated origami stiffness equal to the average experimental value (see Table 1). Performing the same fitting process to the min/max values of the experimentally obtained stiffness of construct C170L gave a confidence interval for α as $0.011 \le \alpha \le 0.04$. The corresponding results for the local nick stiffness are β = 0.009 with a confidence interval $0.0033 \le \beta \le 0.0305$ (Figure 4B). The modeling results suggest that the local stiffness for both HJs and nicks is two orders of magnitude smaller than the one corresponding to the intact double helix. Even though in order to fully understand the origins of this stiffness reduction a systematic study that accounts for all nanoscopic interactions is required, we attribute it mainly to the differences of the mechanical behavior between ssDNA and dsDNA. At the force regime examined here (10–25 pN/helix), the duplexes are in the enthalpic elasticity region and thus fairly stiff (52). However, at the same force levels, the short segments of ssDNA in nicks and at HJs, are still in the entropic regime with an extremely low apparent stiffness (52). This would mean that the tensile loads are able to eliminate any stacking interactions between bases, thus making the nicked helix to behave locally as ssDNA under low entropic forces.

We next used the same modeling framework to understand how the local loss of axial stiffness from HJs and nicks correlates to the macroscopic stiffness of assemblies with different number of neighboring helices. We performed tensile simulations on origami beams with cross-sections consisting of 2–16 double helices packed on both honeycomb and square lattices. The force-displacement curves are shown in Figure 4C and the most striking result is that almost all origami are less stiff than the intact (i.e. having no nicks) double helix. It is also interesting, and counter-intuitive, to note that the relation between the apparent stiffness *k* of each rod and the number *N* of the helices is nonlinear. This can be easily seen in Figure 4D where, for example, the 6-helix rod seems to be slightly stiffer than the 2-helix one, but much less stiff than the 9- and 10-helix bundles. The same figure also shows that in order to reach values of axial stiffness attained by dsDNA, an assembly of at least 16 helices is required.

These results are striking in showing that despite the fact that adding helices in DNA origami increases their overall bending rigidity and the corresponding persistence length, it decreases their apparent axial stiffness. This is crucial for the design and use of DNA nanostructures in cell mechanics studies as traction force meters, and in bio-sensing applications, where DNA nanostructures are subjected to tensile stress imposed by fluid flow (53–57).

## CONCLUSION

In summary, we have designed and synthesized DNA nanobeams with controlled structural features, namely the density of nicks and HJs, two parameters that are key in DNA nanotechnology. Flow-induced stretching experiments indicate that DNA helices with nicks and HJs subjected to axial loads in the 15–25 pN/helix regime are locally very compliant, which in turn drastically reduces the overall apparent stiffness of the nanostructure. Coarse-grained numerical models of continuum rods, that were calibrated using part of the experimental data, were subsequently shown to accurately predict the stiffness of our origami designs as well as the tensile response of other rod assemblies reported in the literature. The same models revealed that the local stiffness for both HJs and nicks is two orders of magnitude less than the corresponding one of the double-helix. Furthermore, by increasing the number of helices in the assembly produces a counter-intuitive nonlinear increase of stiffness. However, our numerical models indicate that a very large number (>16) of helices would be required to recover the stiffness of intact dsDNA.

Our findings are of critical importance for the mechanics-based design of nanoscopic sensors, which requires a complete mechanical characterization of origami nanostructures under general loading conditions that involve coupling of tension, bending and torsion. This study is a first step towards this direction. Furthermore, our experimental measurements provide data needed for the development of multiscale computational models with the ability to reproduce the behavior of DNA origami without any calibrating parameters. Finally, we expect to inspire more studies on establishing the connection between all design aspects of nanostructures and the corresponding mechanical properties. This is a crucial step to reduce over-engineering, minimize unnecessary complexity and thus enable the synthesis of robust higher-order DNA nanostructures.

## Supplementary Material

gkaa985_Supplemental_Files
