# Supplementary material for: Stretching DNA origami: effect of nicks and Holliday junctions on the axial stiffness
Source: Nucleic Acids Res. 2020 Nov 5;48(21):12407–14. doi: 10.1093/nar/gkaa985 (PMC7708044; doi:10.1093/nar/gkaa985)
Supplement: gkaa985_Supplemental_Files [file gkaa985_supplemental_files.zip › NAR-Supplementary Information.pdf]

Supplementary Information

Stretching DNA Origami: Effect of Nicks and Holliday Junctions on the Axial Stiffness

Wei-Hung Jung[1,2,3], Enze Chen[4], Remi Veneziano[5,6], Stavros Gaitanaros[4,*], Yun Chen[1,2,3,*]

[1] Department of Mechanical Engineering, Johns Hopkins University

[2] Institute for NanoBioTechnology, Johns Hopkins University

[3] Center for Cell Dynamics, Johns Hopkins University

[4] Department of Civil and Systems Engineering, Johns Hopkins University

[5] Department of Bioengineering, George Mason University

[6] Institute for Advanced Biomedical Research, George Mason University

* To whom correspondence should be addressed. Tel: +1 (410) 516-5194; Email: yun.chen@jhu.edu

Correspondence may also be addressed to Stavros Gaitanaros. Tel: +1 (410) 516-6482; Email: stavrosg@jhu.edu

DESCRIPTION OF SUPPLEMENTARY INFORMATION

Supplementary File 1

The file contains CaDNAno design for the C85 and C170 structures.

Supplementary File 2

The file contains all the staple sequences used in folding the C85 and C170 structures.

Movie 1

A representative timelapse movie at the left panel shows that the centroid (red circle) belonging to micron-sized particle bound to the C170L DNA nanobeam moved as the DNA nanobeam stretched along the direction of the flow at various rates, imposing different magnitudes of stretching forces. Scale bar: 1 μm. Timestamp format is mm:ss. The corresponding displacement of the DNA nanobeam-bound particle is shown dynamically in the right panel.

SUPPLEMENTARY MATERIAL AND METHODS

Preparation of dsDNA for stretching

The lambda DNA (New England Biolabs, N3011S) was mixed with a 100-fold molar excess of the desired insert oligo 5'-GGG CGG CGA CCT-Biotin-3' and 5'-AGG TCG CCG CCC- Digoxigenin -3' for conjugation of biotin and digoxigenin and at the two ends of the lambda DNA. The mixture was then heated to 70° for 15 minutes to linearize any circular-forms, follow by slowly cooling down from 70 °C to 4 °C for 2 hours at a rate of – 0.5 °C per minute. Unbound oligos were removed using Biorad PCR Kleen purification system. The mixture was then treated by T4 DNA ligase (NEB, M0202S) to repair nicks at 16 °C for 2.5 hours in 50 µL of 1X T4 DNA ligase buffer (50 mM Tris-HCl, 10 mM MgCl2, 10 mM DTT, and 1 mM ATP, pH 7.5) in PCR tubes. The T4 DNA ligase was then inactivated at 65°C for 10 minutes and keep at 4 °C until further usage. The same flow program, chamber, and DNA immobilization approach used in stretching DNA origami were then implemented for stretching dsDNA.

Melting of the DNA origami

qPCR was used to determine the melting temperature (Tm) of the DX-tile beam. The DX-tile beam structures were prepared in the folding buffer at a concentration of 60 nM in a final volume of 15 µL. SYBR Green I nucleic acid stain (Thermofisher, 10,000X initial concentration) was added at a final concentration of 2X. Melting curves were obtained using the Q device from Quantabio with a melting ramp ranging from 40°C to 95°C at a rate of 0.3°C per second. Fluorescence intensity was acquired at every temperature increase step. The first negative derivative of fluorescence as a function of temperature [$-df/dt = f(T)$] was plotted to determine Tm values.

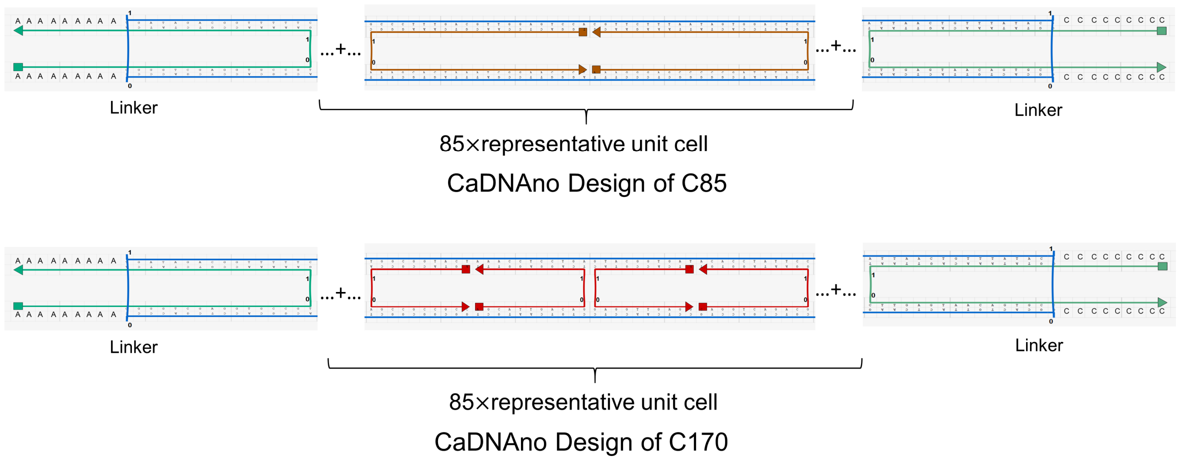

Supplementary Figure S1. Representative designs for staple and linker strands of the DNA constructs.

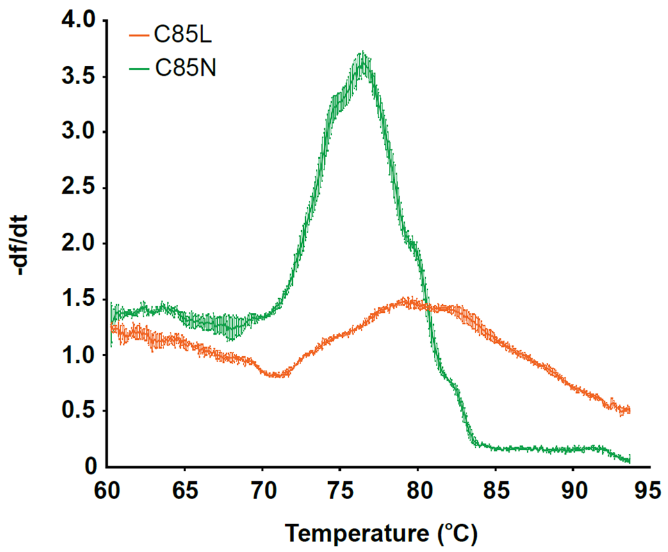

Supplementary Figure S2. Unfolding of the DX-tile beams C85N and C85L as a function of temperature. The melting temperatures (Tm) for both C85N and C85L were determined using qPCR melting experiments. The Tm determined from the curves for C85N and C85L are about 76.0°C and 80.5°C respectively (N= 3).

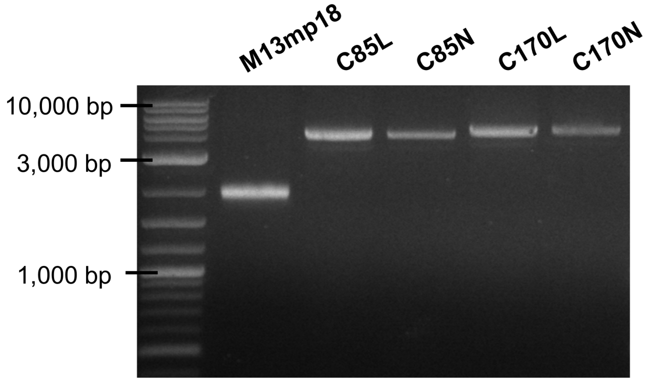

Supplementary Figure S3. Electrophoresis confirmed proper folding of C85L, C85N, C170L and C170N, which exhibited doubled molecular weight compared to the scaffold DNA (m13mp18).

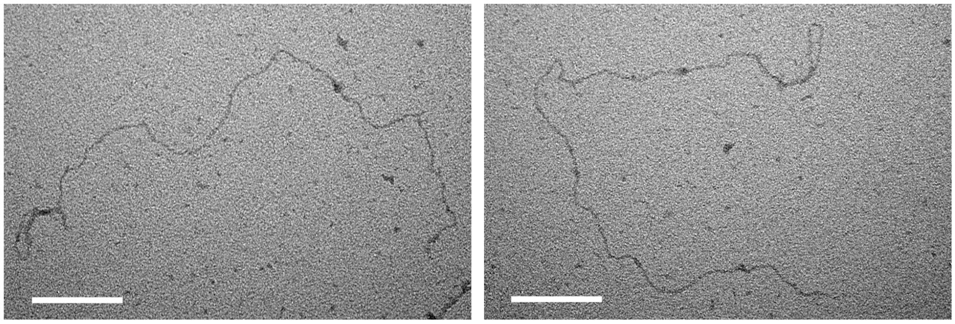

Supplementary Figure S4. TEM images showed that the length of the C170L nanobeams were approximately 1.2 μm, as expected from the design. Scale bar: 100 nm

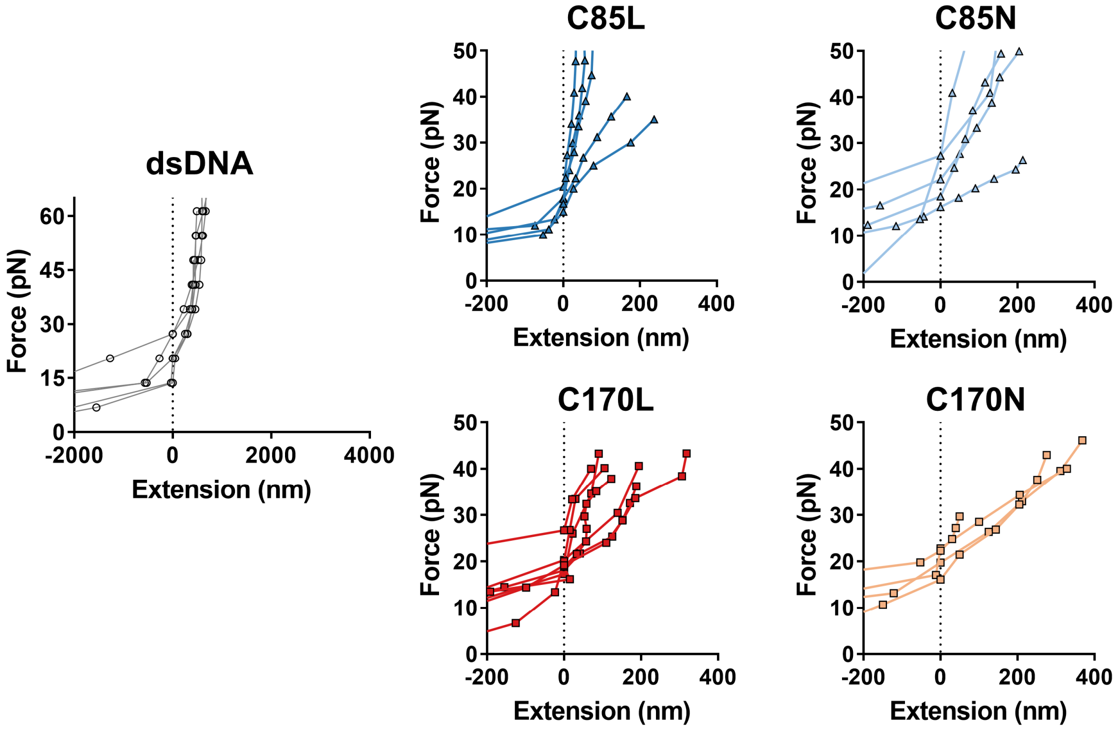

Supplementary Figure S5. All the force-displacement responses of dsDNA (lambda DNA, N = 5), C85L (N = 5), C85N (N = 5), C170L (N =7) and C170N (N = 4) are compared. The extension is defined as the length of the stretched DNA subtracted by the length of straight but unstretched DNA.
